# Stress Sensitivity Is Associated with Differential Accumulation of Reactive Oxygen and Nitrogen Species in Maize Genotypes with Contrasting Levels of Drought Tolerance

**DOI:** 10.3390/ijms161024791

**Published:** 2015-10-19

**Authors:** Liming Yang, Jake C. Fountain, Hui Wang, Xinzhi Ni, Pingsheng Ji, Robert D. Lee, Robert C. Kemerait, Brian T. Scully, Baozhu Guo

**Affiliations:** 1 United States Department of Agriculture, Agricultural Research Service (USDA-ARS), Crop Protection and Management Research Unit, Tifton, GA 31793, USA; E-Mail: yanglm@uga.edu; 2 Department of Plant Pathology, University of Georgia, Tifton, GA 31793, USA; E-Mails: jfount1@uga.edu (J.C.F.); huixu@uga.edu (H.W.); pji@uga.edu (P.J.); kemerait@uga.edu (R.C.K.); 3 School of Life Sciences, Huaiyin Normal University, Huaian 223300, China; 4 United States Department of Agriculture, Agricultural Research Service (USDA-ARS), Crop Genetics and Breeding Research Unit, Tifton, GA 31793, USA; E-Mail: xinzhi.ni@ars.usda.gov; 5 Department of Crop and Soil Sciences, University of Georgia, Tifton, GA 31793, USA; E-Mail: deweylee@uga.edu; 6 United States Department of Agriculture, Agricultural Research Service (USDA-ARS), U.S. Horticultural Research Laboratory, Fort Pierce, FL 34945, USA; E-Mail: brian.scully@ars.usda.gov

**Keywords:** maize seedlings, drought stress, reactive oxygen species, reactive nitrogen species

## Abstract

Drought stress decreases crop growth, yield, and can further exacerbate pre-harvest aflatoxin contamination. Tolerance and adaptation to drought stress is an important trait of agricultural crops like maize. However, maize genotypes with contrasting drought tolerances have been shown to possess both common and genotype-specific adaptations to cope with drought stress. In this research, the physiological and metabolic response patterns in the leaves of maize seedlings subjected to drought stress were investigated using six maize genotypes including: A638, B73, Grace-E5, Lo964, Lo1016, and Va35. During drought treatments, drought-sensitive maize seedlings displayed more severe symptoms such as chlorosis and wilting, exhibited significant decreases in photosynthetic parameters, and accumulated significantly more reactive oxygen species (ROS) and reactive nitrogen species (RNS) than tolerant genotypes. Sensitive genotypes also showed rapid increases in enzyme activities involved in ROS and RNS metabolism. However, the measured antioxidant enzyme activities were higher in the tolerant genotypes than in the sensitive genotypes in which increased rapidly following drought stress. The results suggest that drought stress causes differential responses to oxidative and nitrosative stress in maize genotypes with tolerant genotypes with slower reaction and less ROS and RNS production than sensitive ones. These differential patterns may be utilized as potential biological markers for use in marker assisted breeding.

## 1. Introduction

Drought stress dramatically limits crop growth and development, and can trigger a significant decrease in crop yield and quality. This is especially evident for maize grown as a summer crop in the Southern U.S. as drought stress in combination with high temperatures aggravate stress severity, and exacerbate *Aspergillus flavus* colonization leading to pre-harvest aflatoxin contamination [1,2,3]. Therefore, tolerance and adaptation to drought stress is an important trait of crops, and a detailed understanding of maize responses to drought stress is of importance for crop breeding and sustainable agriculture.

Mild oxidative or nitrosative stress affects signal transduction pathways and induces gene expression [4]. More drought-related factors can disturb the normal metabolic homeostasis of crop plants producing visible foliar symptoms and impairing growth by altering the physiological, biochemical and molecular statuses of plants. Plants can adapt to drought stress by regulating the homeostasis of many biochemical pathways related to water transport, transpiration, osmotic balance, signal transduction, antioxidant mechanisms, and the protection or degradation of proteins [1,5,6,7]. One of the earliest events in plant drought responses is a burst of reactive oxygen species (ROS) production leading to the over-accumulation of superoxide radicals (O_2_·^−^) and hydrogen peroxide (H_2_O_2_) [8,9]. Despite the damaging potential of these molecules, ROS are continuously produced at a low level by some metabolic processes in plants [10]. These ROS also act as signaling molecules to regulate the expression of multiple genes and diverse stress-responsive pathways [11]. However, over-accumulation of these ROS can change the normal redox status in plants under stress conditions, and initiate oxidative damage to proteins, DNA, and lipids, ultimately leading to destruction of macromolecules and can induce the cell death in plant leaves [12,13,14,15,16]. Plant defense systems can provide enough protection against ROS damage produced at normal growth conditions through enzymatic and non-enzymatic antioxidant systems [17]. Previous studies have shown that these antioxidant systems can be induced by water stress accompanied with increasing activities of superoxide dismutase (SOD) and catalase (CAT) [18,19]. However, the generation of higher levels of ROS may destroy or overwhelm the defensive capabilities of these systems resulting in oxidative stress, and further exacerbation of visible drought symptom severity [12,20].

Nitric oxide (NO) is an additional redox signal that can react with some ROS to form highly reactive molecules referred to as reactive nitrogen species (RNS). These RNS can trigger various physiological processes [21,22]. Recent studies have reported that environmental stress factors including cold, heavy metal, and salt stresses can promote NO production [23,24,25], and modulate the level of gene expression and enzyme activities of RNS metabolism components [22,26,27]. Nitric oxide can also enhance plant drought tolerance by regulating the leaf photosynthetic rates, relative water content, and antioxidant systems [28,29,30]. Moreover, Wang *et al*. [31] found that NO resulting from abscisic acid (ABA) signaling resulted in the *S*-nitrosylation of protein kinase SnRK2.6 that can further result in stomatal closure to reduce transpirational water loss.

As signal molecules, H_2_O_2_ and NO have a synergistic effect in response to drought stress. Liao *et al*. [32] reported that drought stress can be alleviated by dose-dependent NO and H_2_O_2_ in marigold explants and promote adventitious root development by improving photosynthetic performance and regulating carbohydrate and nitrogen accumulation. It has also been demonstrated that H_2_O_2_ can increase NO in antioxidant defense processes by activating mitogen-activated protein kinase (MAPK) cascades in maize [33]. Although stress conditions have the common effect of resulting in the accumulation of H_2_O_2_ and NO in plants, the biological response employed in maize plants can be different depending on the genotype of plants, and the intensity and duration of drought stress. However, the detailed molecular mechanisms through which they work are only partly understood.

Previous research has demonstrated that maize drought tolerance is associated with different responsive patterns in the processes of redox homeostasis and ROS metabolism in kernels of maize lines possessing differing drought sensitivities. For example, moderate drought stress triggers increases in the expression of ROS-scavenging enzymes such as SOD, glutathione S-transferase (GST), and antioxidant enzymes such as thioredoxin and peroxiredoxin in kernels of the sensitive maize genotype B73 as compared to the tolerant genotype Lo964 [19]. Expression profiling of genes encoding the proteins mentioned above have also exhibited genotype-specific responses in drought tolerant and sensitive maize lines in previous studies [34,35,36,37,38,39]. In order to further characterize the impact of drought stress on maize, the time-dependent responsive patterns in physiological and photosynthetic indices, specifically ROS and RNS metabolism components, were monitored in the leaves of six genotypes displaying varying sensitivity to drought stress. It was found that drought stress induced differential patterns of oxidative and nitrosative stress in tolerant and sensitive maize genotypes (Table 1). Given these differential patterns, it is possible that these indices can be utilized as biological markers in marker-assisted breeding to enhance maize drought tolerance and its secondary impact on aflatoxin contamination.

## 2. Results

### 2.1. Seedling Morphological Responses to Drought in Selected Maize Genotypes

To examine the responses of selected maize genotypes to drought stress, the morphological responses of the tolerant lines A638, Grace-E5, Lo964, and Va35 along with the sensitive lines B73 and Lo1016 (Table 1) to drought stress were measured every 3 days after induction (DAI) until 12 DAI for the drought treated and irrigated control samples at the V3-V4 growth stage. The drought stress was applied up to 9 DAI in the treated samples with a watered recovery period from 9 to 12 DAI. As expected, drought stress resulted in a visible loss of turgor with curling and wilting symptoms apparent in seedling leaves during the period of drought, and this phenotype gradually worsened with continuing decreases in soil water content (SWC) over time. The SWC was held consistent in the pots of all six maize lines at each time point throughout the drought treatments (Figure S1). The sensitive lines exhibited more visible symptoms in response to drought treatments with increased flaccidity in seedling leaves from 3 to 9 DAI with the most obvious symptoms observed by 9 DAI, and with some seedlings dying during the course of the study. The tolerant lines, however, exhibited wilting symptoms later than the sensitive lines during the drought treatment.

**Table 1:** Maize genotypes selected in this study.

Genotypes	Pedigree	Origin	Tolerance to Drought	Reference
B73	Recurrent selection population (C5) of Iowa Stiff Stalk Synthetic	Iowa, USA	Susceptible	[40,41,42]
Lo1016	P3369A × Lo876o2	Italy	Susceptible	[37,43,44]
A638	(V3 × Wf9) × Wf9	Minnesota, USA	Moderate	[45]
Lo964	P3183	Italy	Tolerant	[37,43,44]
Va35	(C103 × T8) × T8	Virginia, USA	Tolerant	[46,47]
Grace-E5	–	CIMMYT, Mexico	Tolerant	[48]

CIMMYT, International Maize and Wheat Improvement Center.

Following the irrigated recovery period from 9 to 12 DAI, the tolerant lines displayed relatively stronger growth recovery from drought stress with only about half of the sensitive line plants survived. The growth rates of the seedlings were also measured during the course of the experiment. The growth rates up to 3 DAI were higher than those observed at 6 and 9 DAI under drought treatment conditions in all the lines (Figure S2). Although the growth rates of the six maize lines showed the same decreasing trend during the progressive water deficit treatment, the growth rates of sensitive lines B73 and Lo1016 reached 0 at 6 DAI, but the other lines stopped growing at 9 DAI (Figure S2). After 3 days of recovery from water deficit stress, the growth rate of all lines remained unchanged. Leaf relative water content (LRWC) was also examined in samples at 2 days prior to drought treatment (noted as “−2 DAI”). The LRWC decreased at 3 DAI, and continued to decrease at 6 and 9 DAI, with the LRWC of the tolerant lines Va35 and Grace E-5 being significantly higher than that of the sensitive lines B73 and Lo1016, especially at 9 DAI (Figure S3).

### 2.2. Changes of Chlorophyll Content and Photosynthesis Parameters in Response to Progressive Drought Stress and Recovery

To determine the influence of drought stress on photosynthetic capacity, leaf chlorophyll content and photosynthetic parameters were investigated. The chlorophyll content of B73 and Lo1016 was significantly lower than that observed in the other four lines under the non-stressed condition (Figure S4). Although chlorophyll content was decreased following the drought treatments for the six maize lines, the chlorophyll content of stressed B73 and Lo1016 leaves was significantly lower than that of moderately tolerant and tolerant lines at 3 and 6 DAI. The tolerant lines and A638 have higher chlorophyll content under drought stress than the susceptible lines under drought or irrigated conditions at 9 DAI, and stress recovery for 3 additional days did not result in an obvious increase in chlorophyll content (Figure S4).

**Figure 1:**
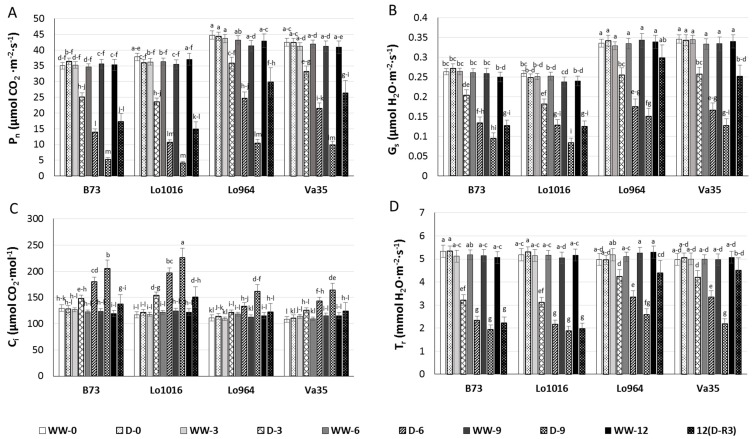
Photosynthetic parameters of seedling leaves from the sensitive genotypes, B73 and Lo1016, and the tolerant ones, Lo964 and Va35, under well-watered (WW) and drought stressed (D) conditions. Photosynthetic metrics were measured in sensitive and tolerant genotypes during drought stress and post-recovery including P_n_ (**A**), G_s_ (**B**), C_i_ (**C**), and T_r_ (**D**) measured from 9:00 to 11:00 in the morning for every collection time, at day zero (0), 3, 6, 9 or 12, on young leaves. Different letters indicate significant differences (*p* < 0.05) based on Tukey’s test between control and treatments and between different treatment times. Data represent the mean ± SD of three or more replicates.

Four maize lines, including sensitive lines B73 and Lo1016 and tolerant lines Lo964 and Va35, were selected to investigate their photosynthesis parameters in leaves, soil water deficit decreased the leaf photosynthesis rate (P_n_) (Figure 1A). The influence of drought stress on P_n_ in B73 and Lo1016 was more rapid than that of Lo964 and Va35. In B73 and Lo1016, P_n_ decreased by 85.3% and 88.4%, respectively, by 9 DAI relative to the well-watered controls while P_n_ had decreased by 74.9% and 76.0% in Lo964 and Va35, respectively. After 3 days of stress recovery (12 DAI), P_n_ was restored to only 49.0% and 40.3% of the level of the non-stressed controls for B73 and Lo1016, respectively, compared to 69.4% and 65.2% in Lo964 and Va35.

A similar pattern occurred in the stomatal conductance (G_s_) of the sensitive and tolerant lines in response to drought stress (Figure 1B). The P_n_ and G_s_ for Lo964 and Va35 were higher than those observed in B73 and Lo1016 throughout the water deficit treatment and stress recovery periods. Intercellular CO_2_ concentration (C_i_) increased with drought treatment, and the highest C_i_ was observed for drought-treated Lo1016 and B73 at 9 DAI, which had a larger increase in C_i_ in the drought treatment in comparison to their respective well-watered controls in contrast to Lo964 and Va35 which showed a slight increase during the progressive drought treatment (Figure 1C). However, all the selected lines did not show a marked difference in C_i_ following stress recovery. In addition, the drought stress treatment significantly decreased the transpiration rate (T_r_) in all the six lines during progressive water deficit stress (Figure 1D). However, B73 and Lo1016 showed a rapid initial decrease in T_r_ while Lo964 and Va35 showed a gradual, progressive decrease in T_r_ during the process of water deficit treatment.

### 2.3. Drought Responses of Abscisic Acid (ABA) and Indole-3-Acetic Acid (IAA) Contents

**Figure 2:**
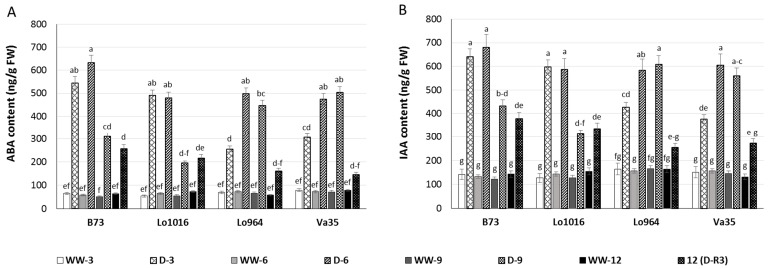
ABA and IAA content in maize seedling leaves under well-watered (WW) and drought (D) conditions. Phytohormone levels were measured in sensitive and tolerant genotypes over drought stress and recovery including ABA (**A**); and IAA (**B**). Different letters indicate significant differences (*p* < 0.05) based on Tukey’s test between control and treatments and between different treatment times. Data represent the mean ± SD of three or more replicates.

Abscisic acid (ABA) and indole-3-acetic acid (IAA) are important phytohormones in plant responses during drought stress and development [49,50], therefore, subsequent changes of ABA and IAA contents in maize seedling leaves under drought stress were determined. Drought-stressed maize leaves exhibited higher ABA content than ones under well-watered conditions. At 3 and 6 DAI, drought-stressed B73 and Lo1016 presented a sharp rise in ABA contents, then a significant decrease with ongoing stress compared to the well-watered controls (Figure 2A). However, the ABA contents displayed a progressively increasing trend for Lo964 and Va35 during continued drought induction, and recovery did not reduce ABA contents to the level of well-watered controls (Figure 2A). Drought stress induced an increase of IAA content in maize seedling leaves. IAA contents gradually increased at 3 to 9 DAI of drought stress, and drought recovery decreased the IAA contents close to the level of well-watered plants in the tolerant lines (Figure 2B). For B73 and Lo1016, IAA contents increased dramatically at 3 to 6 DAI, and then decreased at 9 DAI, but IAA levels did not recover to the level of well-watered plants (Figure 2B).

### 2.4. Effect of Drought Stress on ROS Metabolism

**Figure 3:**
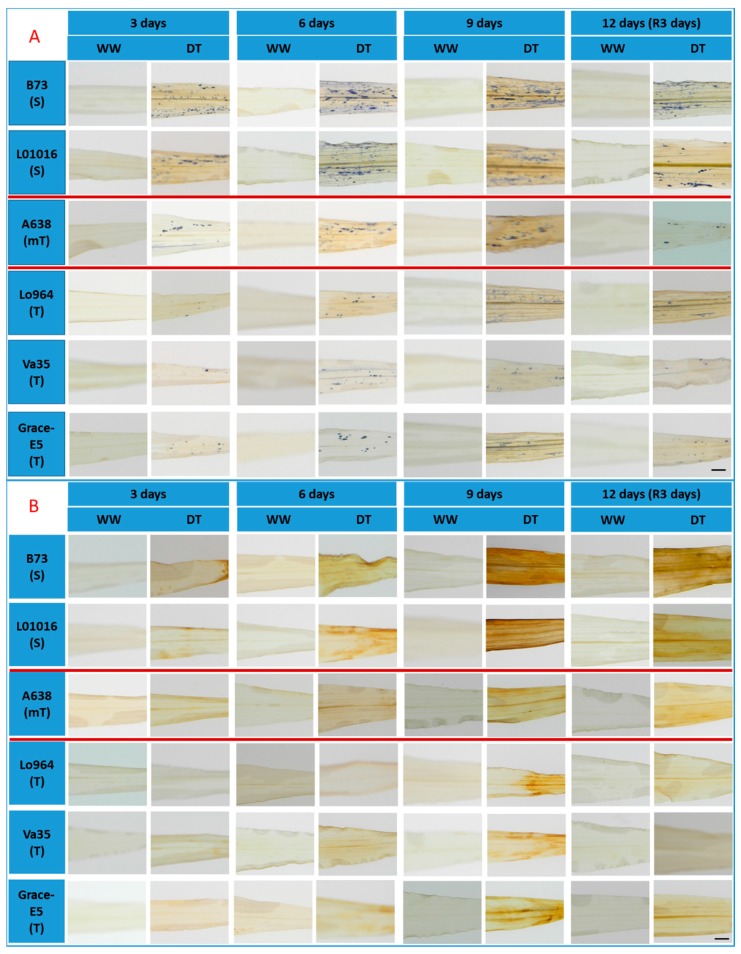
Visualization of superoxide radical and hydrogen peroxide in the leaves of maize plants under well-watered (WW) and drought stressed (DT) conditions. Endogenous O_2_**^·^**^−^ levels were monitored by staining O_2_**^·^**^−^ using a Nitro blue tetrazolium (NBT) staining method (**A**); and the endogenous H_2_O_2_ level was monitored by staining H_2_O_2_ using 3,3′-diaminobenzidine tetrahydrochloride hydrate (DAB) (**B**). Scale bar in (**A**,**B**), 5 mm.

Detection of O_2_**^·^**^−^ through the use of Nitroblue Tetrazolium Chloride (NBT) staining indicated that B73 and Lo1016 accumulated much more O_2_**^·^**^−^ than A638, Lo964, Va35, and Grace E-5 over time in response to drought stress (Figure 3A). Although recovery decreased the content of O_2_**^·^**^−^ in the leaves of B73 and Lo1016, the O_2_**^·^**^−^ quantities were still higher than that of A638, Lo964, Va35, and Grace E-5 (Figure S5A). Specifically, B73 and Lo1016 accumulated approximately 2.5 times more O_2_**^·^**^−^ in their leaves in comparison to Lo964 and Va35 (Figure S5A). Time-course detection of H_2_O_2_ accumulation using 3,3′-diaminobenzadine (DAB) staining in the leaves revealed that no obvious changes in well-watered controls, whereas H_2_O_2_ content increased progressively in drought-induced leaves over time with B73 and Lo1016 accumulating more H_2_O_2_ than Lo964, Va35 and Grace E5 which is consistent with microscopy observations. Also, the moderately resistant line A638 showed more H_2_O_2_ accumulation than Lo964, Va35 and Grace E5, but less than B73 and Lo1016 (Figure 3B and Figure S5B). These results showed that stress occurred in the leaves of maize seedlings under drought treatment conditions causing severer oxidative stress for sensitive and moderate lines than for tolerant ones.

**Figure 4:**
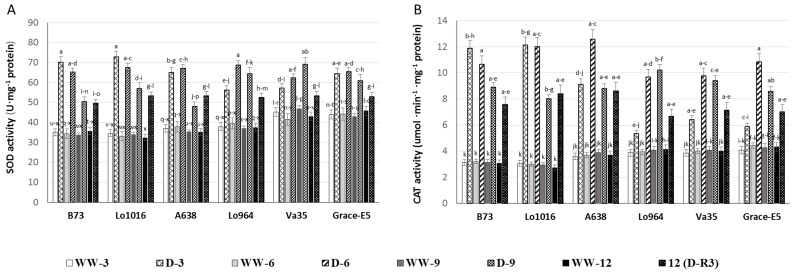
Effects of drought treatments on SOD and CAT activities in maize seedling leaves. The activities of ROS-remediating enzymes were measured in sensitive and tolerant genotypes over drought stress and recovery including SOD (**A**) and CAT (**B**). Different letters indicate significant differences (*p* < 0.05) based on Tukey’s test between control and treatments and between different treatment times. Data represent the mean ± SD of three or more replicates.

The activities of two key antioxidant enzymes, superoxide dismutase (SOD) and catalase (CAT) were measured to determine their roles in defense during drought treatments. It was found that the antioxidant enzyme activities were enhanced in response to oxidative stress in the examined drought stressed maize lines (Figure 4A,B). Specifically, SOD activity in the leaves of B73 and Lo1016 increased by 2-fold immediately after drought treatment for 3 days then decreased gradually at 6 and 9 DAI. However, SOD activity increased by 1.3, 1.6, and 1.6 folds in Lo964 and Va35 at 3, 6 and 9 DAI, respectively, displaying a progressive increasing trend. But, SOD activities in A638 and Grace E-5 were induced at a high level at 3 and 6 DAI, and then decreased at 9 DAI (Figure 4A). The CAT activities displayed a rapid increase and then a slight drop after drought treatment for 3, 6 and 9 DAI by 3.4, 3.6 and 2.7 folds in B73 and Lo1016. The remaining lines displayed a gradually increasing pattern in CAT activities (Figure 4B). Although drought recovery reduced the activities of SOD and CAT, they remained higher in the drought stressed plants than in the well-watered controls.

### 2.5. NO Production and RNS-Related Enzyme Activities during Drought Stress

**Figure 5:**
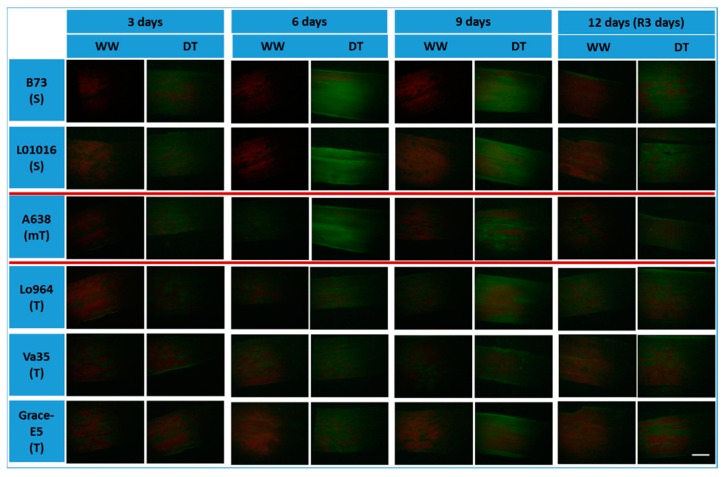
Visualization of nitric oxide (NO) in maize leaf tissues subjected to drought stress. Endogenous NO levels was monitored in maize leaves by staining NO using DAF-FM diacetate, and displayed by green fluorescence; the red fluorescence represents the chlorophyll intensity. WW refers to well-watered leaves; DT refers to drought treated leaves of all tested maize genotypes. Scale bar, 5 mm.

An NO-specific fluorescent probe, 4-amino-5-methylamino-2ʹ,7ʹ-difluorofluorescein diacetate (DAF-FM DA), was used to monitor the endogenous NO production in the leaves of the six maize lines. Significant changes in NO content were observed between lines with contrasting drought sensitivity at 3 and 9 DAI and 3-day recovery (Figure 5). Normal irrigated plants had the weak NO fluorescence during 12-day period, but drought stress resulted in an increase of NO fluorescence intensity. Significant increases in NO accumulation were observed at 3 and 6 DAI with the most significant NO accumulation observed at 6 DAI. Slight decreases were also observed at 9 DAI in the leaves of B73 and Lo1016 and moderately tolerant A638. However, drought stress induced a gradual increase in NO accumulation from 3 to 9 DAI in Lo964, Va35 and Grace E-5 (Figure 5).

Quantitative analyses showed that the intensity of NO accumulation in the leaves of well-watered control maize plants were relatively constant during the 12-day period. An early burst of NO at 3 DAI was around 1.5-fold higher in the sensitive lines B73 and Lo1016 and moderately tolerant A638 than in the tolerant lines Lo964, Va35 and Grace E-5, and then reached to about 2.1-fold higher at 6 DAI. The NO content displayed a gradually increased trend in the tolerant lines Lo964, Va35 and Grace E-5 in response to drought, though not as much as determined in the sensitive lines B73 and Lo1016 and moderately tolerant A638 (Figure S6). This increasing trend of NO accumulation is consistent with that of O_2_**^·^**^−^ and H_2_O_2_ in B73 and Lo1016 under drought stress. Lo964, Va35 and Grace E-5 illustrated the same response profiles in NO, O_2_**^·^**^−^ and H_2_O_2_ accumulation under drought treatment conditions. For the moderately tolerant line A638, the response pattern of gradual increasing at 3–6 DAI and slight decreasing at 9 DAI for NO accumulation is different from the gradually increasing trend at 3–9 DAI for O_2_**^·^**^−^ and H_2_O_2_ under drought stress conditions (Figure 3 and Figure 5).

**Figure 6:**
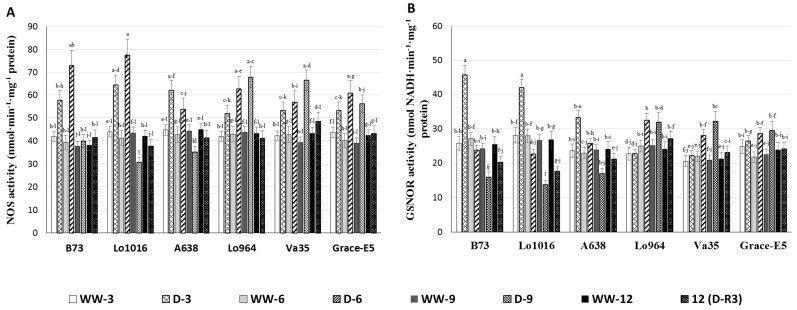
The NOS and GSNOR activities in maize seedling leaves under well-watered (WW) and drought (D) conditions. Nitrosative stress-related enzyme activities were measured in sensitive and tolerant genotypes over drought stress and recovery including NOS (**A**); and GSNOR (**B**). Different letters indicate significant differences (*p* < 0.05) based on Tukey’s test between control and treatments and between different treatment times. Data represent the mean ± SD of three or more replicates.

Nitric oxide synthase (NOS) serves as a key enzyme for NO biosynthesis [51,52]. To examine the contribution of NOS to the drought-induced NO burst previously observed in the maize seedling leaves, we measured NOS activity in the lines during drought stress. NOS activities were found to vary significantly among the lines under drought stress (Figure 6A). The lines B73 and Lo1016 had significantly higher NOS activities than Lo964, Va35 and Grace E-5 at 3 and 6 DAI. Drought treatment triggered significantly elevated NOS activity at 3 and 6 DAI in B73 and Lo1016, 1.5- and 2.0-fold, respectively, which then reduced to levels similar to the well-watered controls at 9 DAI. In contrast, the NOS activities in Lo964, Va35 and Grace E-5 exhibited a slight increasing trend after drought treatment. After stress recovery, NOS activities declined to levels similar to those of the well-watered controls (Figure 6A).

*S*-nitrosoglutathione reductase (GSNOR), as a RNS-scavenging enzyme, can control NO levels, regulate *S*-nitrosylation of proteins by modifying cysteine residues using NO, and reduce the content of *S*-nitrosoglutathione (GSNO) [22,53]. The GSNOR activity in drought treated B73, Lo1016, and A638 increased rapidly up to 1.9, 1.5 and 1.3 folds higher than that of the well-watered controls at 3 DAI, respectively, then dropped to below the levels of the well-watered controls at 6 and 9 DAI. Three days after a recovery irrigation, GSNOR activities were not restored to normal levels found in the controls (Figure 6B). Conversely, GSNOR activities in Lo964, Va35, and Grace E-5 did not display significant changes at 3 DAI, then exhibited slight and gradual increasing profiles at 6–9 DAI compared to the controls, and 3-day recovery decreased the GSNOR activities to the same level as that of well-watered plants.

### 2.6. Physiological and Biochemical Patterns Allow Clear Separation of Drought Tolerance Characterization between Different Maize Lines

**Figure 7:**
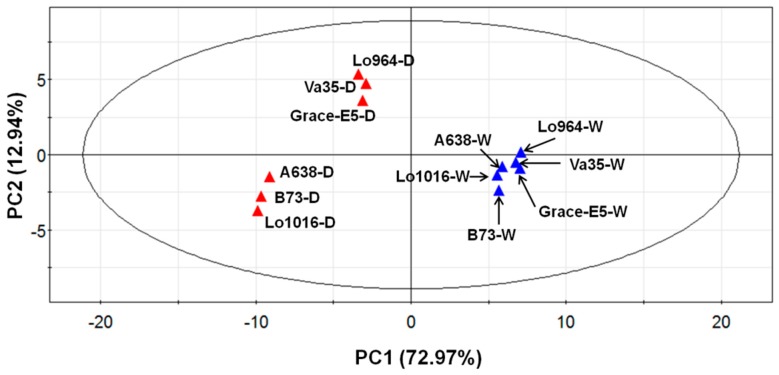
Principal component analysis (PCA) of all physiological and biochemical data in six different lines under well-watered and drought treated conditions. The blue points represent the well-watered (W) plants, while the red points represent the drought-stressed (D) plants.

Subjecting the physiological and biochemical data to a principal component analysis (PCA) separated the lines relative to all indices, independent from genotype or treatment influences (Figure 7), indicating significant differences exist within the physiological and biochemical indices between the different lines. PC1 (73% variance) separates the different treatments (well-watered and drought stress conditions), and shows that drought-treated lines display obviously different stress response patterns in comparison to well-watered ones with the lines divided into two groups. The first group includes Lo964, Va35, and Grace E5; and the second group includes B73, Lo1016, and A638. PC2 (13% variance) separates the different reactions of all lines responding to drought stress, and shows that Lo964, Va35, and Grace E5 have contrasting drought tolerance levels in comparison with B73, Lo1016, and A638.

Under well-watered conditions, all the lines show very similar responsive patterns, while under drought-treatment conditions the lines exhibited more diverse physiological and biochemical changes with regard to their genetic background (Figure 7 and Figure S7). The PCA analysis also separated lines predicted to be drought-sensitive or tolerant, and indicated that the factor that influenced the measured traits most was stress treatment, followed by genotype. In addition, the separation due to drought treatment was most profound at 6 and 9 DAI compared to 3 DAI (Figure S7).

### 2.7. Correlation and Variance Analysis of All Tested Traits in All Lines

**Figure 8:**
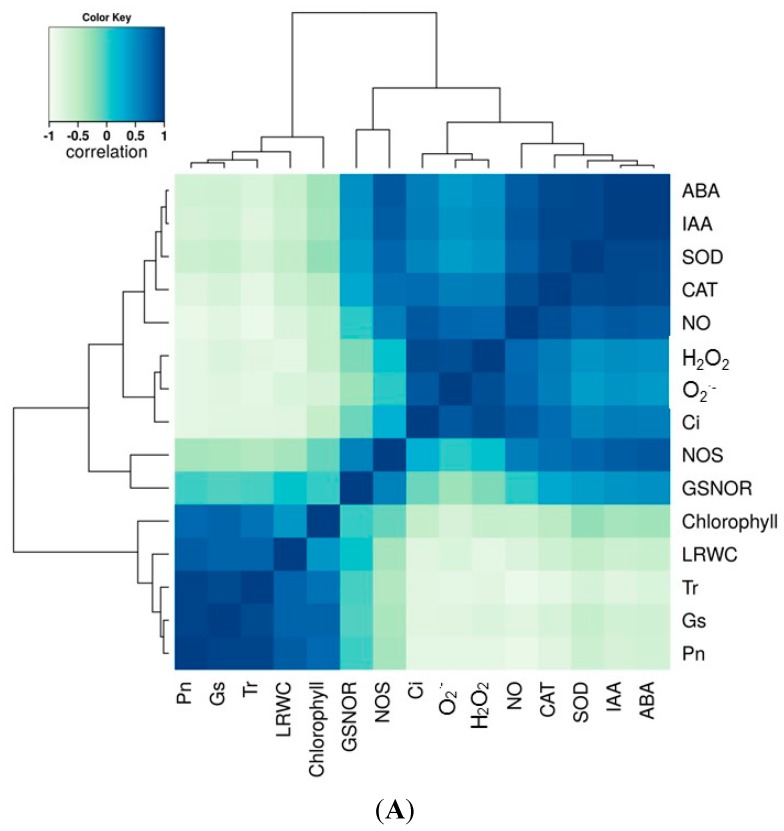

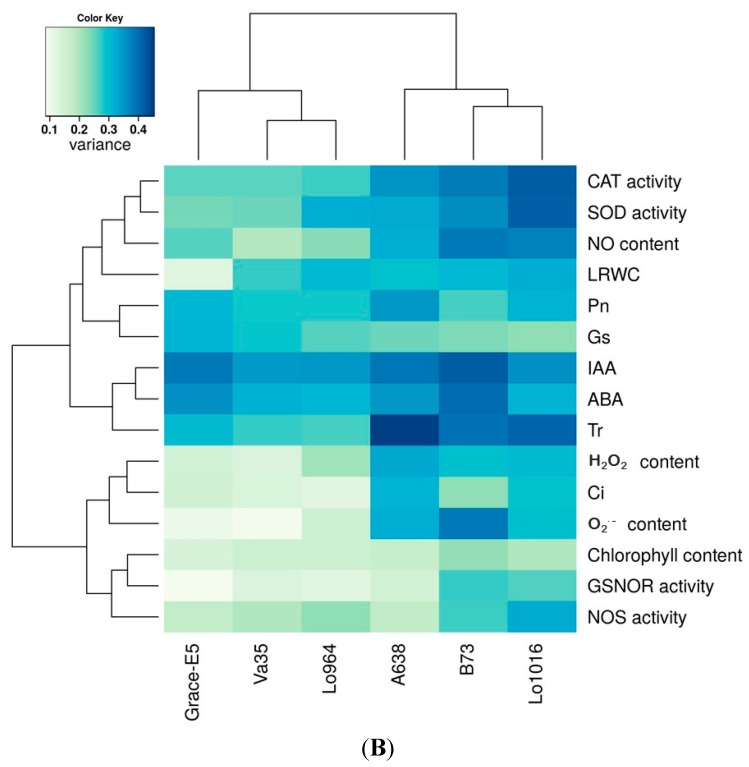
Correlation and variance analysis of all tested dataset in six tested lines under well-watered and drought treated conditions. (**A**) shows the correlation among each parameter determined; and (**B**) shows the variance among each trait and each sample during the period of drought treatments.

In this study, we sought to correlate the physiological responses of the maize lines during drought stress with hormone content and biochemical processes involved in ROS and RNS metabolism. Therefore, a correlation analysis of all the tested parameters was performed and as shown in Figure 8. From the correlation matrix heat map, a positive correlation between hormone accumulation and RNS metabolism and ROS-scavenging enzyme activities was observed (Figure 8A). A positive correlation between G_s_, NO, and ROS was also observed. Photosynthesis parameters P_n_, G_s_, and T_r_ also positively correlated with chlorophyll content and LRWC. A strong negative correlation for the photosynthesis parameters P_n_, G_s_, and T_r_ with the ROS and RNS systems was observed, and GSNOR negatively correlated with the ROS components O_2_·^−^ and H_2_O_2_ (Figure 8A). Analysis of the variance showed that drought treatment and variety significantly contributed to the variance observed in the physiological and biochemical traits (Figure 8B). The variation in ROS content and activities of ROS-scavenging enzymes were highly significant among maize genotypes under drought stress conditions and water recovery; however, less variation was recorded in chlorophyll content and GSNOR activity.

Time-series hierarchical clustering analysis of each tested trait separates all six lines into two main clusters which is consistent with the lines’ corresponding sensitivity to drought stress. Cluster one includes drought-sensitive lines B73 and Lo1016, and moderate A638; the second cluster contains the drought-tolerant lines Lo964, Va35 and Grace E5, with the exception of chlorophyll content and LRWC (Figure S8). This result is supported by the PCA analysis as shown in Figure 7, and consistent with the analysis of variance shown in Figure 8B. As expected, NO and its synthetic enzyme NOS exhibit the same clustering patterns to all lines, showing their close correlation in NO metabolism.

## 3. Discussion

### 3.1. Morphological and Physiological Responses to Drought Stress

Continuous drought stress resulted in significant growth inhibition in all tested lines, with the effect being much more pronounced in B73, Lo1016 and A638, and the drought tolerance of these six lines is consistent with previous reports determined through the observation of morphological responses to drought stress (Table 1). Photosynthetic parameters were affected by drought treatment in all tested lines, decreases in P_n_, G_s_ and T_r_ and increase in C_i_ values may imply that the plant is subjected to stress conditions [54,55], and it can be proposed that the inhibition of photosynthesis was caused by the hypersensitive early stomata closure, with more pronounced occurrence in sensitive genotypes [56]. In addition, higher C_i_ but lower photosynthetic rate may be caused by non-stomatal factor [55,57]. The morphological injury and growth inhibition caused by drought stress is transmitted by a hormone signals such as ABA and IAA which are induced during drought [37,58,59]. A rapid induction of ABA and IAA in leaves of maize seedlings was found under drought stress conditions, with more rapid and higher increases observed in the sensitive genotypes in comparison to the tolerant ones, which was similar with the results in Lo1016 and Lo964 under PEG-induced stress [37]. Taken together, these results imply that hormone signaling functions during drought stress to regulate photosynthetic processes in order to cope with stress-related damage [60,61]. Plant adaptation to stress can also be regulated through ABA-based activation of ROS signaling [62]. These ROS molecules function as second messengers, and play a rate-limiting role in ABA signal transduction [63,64].

### 3.2. Drought Stress Induces an ROS Burst and Disturbs the Redox Homeostasis

ROS can be transient induced in the early events of plant response to stress, and can be considered to act as secondary message for induction of stress response [65,66]. But excess ROS accumulation can be induced following stress treatment to a certain extent, and damages the plant [67,68]. Drought stress can cause ROS accumulation in plant leaves, and the observed ROS patterns greatly depend on the time and severity of the stress [18,69]. Therefore, it is important to characterize the profiles of ROS accumulation associated with specific drought stress conditions. As expected, drought stress resulted in increases of ROS, specifically O_2_**^·^**^−^ and H_2_O_2_, levels especially in sensitive lines, which accumulate ROS at the early stages of drought treatment, while tolerant lines progressively and gradually accumulate ROS. This indicates that this drought treatment resulted in severe oxidative stress to the sensitive lines and moderate one, but relatively mild stress for the tolerant lines. These observations are supported by the different phenotypic and morphological traits of these maize genotypes under the same drought treatments.

In order to avoid oxidative injury due to ROS accumulation; plants possess complex antioxidant systems to scavenge ROS. The well-known antioxidant enzymes SOD and CAT can catalyze the decomposition of O_2_^−^ and H_2_O_2_; respectively; to prevent the over-accumulation of these ROS. Interestingly; drought treatments resulted in increased CAT and SOD activities in all the tested seedlings likely in response to the over-production of O_2_^−^ and H_2_O_2_ in order to prevent the oxidative injury. It can be suggested that drought stress induced a rapid production of ROS leading to disruption of cellular redox homeostasis and activation of the antioxidative system and ROS-scavenging enzymes [15,66].

In this study, drought stress induced differential responsive profiles of SOD and CAT activities in sensitive and tolerant lines with higher activities and levels being rapidly induced in sensitive lines at the initial stage of drought treatments compared to tolerant ones which displayed gradually increased patterns. Similar phenomena were also found in kernels of B73 and Lo964 subjected to drought stress in our previous research [19]. In addition, drought stress-induced ROS over-production and scavenging enzyme activities were reduced by water recovery, especially in tolerant lines compared to sensitive lines. Similar ROS accumulation and induced increase activities of antioxidant enzymes have been described for other plants. For example, in *Lotus japonicus*, water deficit caused an increase in ROS production accompanied by an increase of antioxidant enzymes [18], and drought has been shown to induce a much stronger oxidative stress in leaves of drought-sensitive varieties of barley compared to tolerant ones [70]. Plant adaptation to drought stress may depend on different response and defense mechanisms to oxidative damage in different genotypes, including the ability to maintain high levels of antioxidant system activity [71].

### 3.3. Drought Stress Affects NO Homeostasis

Drought stress caused an increase in NO production in all tested maize genotypes and was more pronounced in sensitive genotypes. This result implies that NO participates as an important messenger in the defense processes of plants, specifically maize, in responding to drought stress [72]. Similar results relating increased NO content to drought stress responses have been described in leaves of *Poncirus trifoliata* and *Lotus japonicus* [18,28], and under other stress conditions [25,27,73]. It has also proposed that NO is a signaling intermediate in enhancing the tolerance of maize seedlings to drought stress [72]. The NO content response patterns in the leaves is also well correlated with the increase of NOS activities observed during drought treatments in this study. This suggests that NOS activity, as one of the sources of NO, may be up-regulated to generate more NO in response to drought stress. Additionally, increased GSNOR activities were also found in the leaves of all six lines under drought stress. The GSNOR enzyme is a key regulator of NO synthesis and homeostasis [74,75]. Frungillo *et al*. [76] reported that mutation and silencing of *GSNOR*1 caused NO accumulation in *Arabidopsis thaliana*. Hence, it is proposed RNS scavenging systems such as GSNOR are produced in response to over-accumulation of NO in plants, which may result in nitrosative stress, in response to stress conditions.

### 3.4. Drought Stress Responses Involve Interconnections between ROS and RNS

Plants possess effective and fine-tuned mechanisms to regulate the metabolic balance, and to control the levels of all kinds of molecules, including ROS and RNS levels [30]. The results obtained here indicate that drought stress resulted in an imbalance in ROS and RNS metabolism and homeostasis, especially in drought sensitive genotypes. This suggests that drought treatments induce oxidative and nitrosative stress in maize seedlings to different degrees in sensitive and tolerant genotypes. It is known that ROS and RNS metabolism are closely connected and cross-reacted, and that ROS and NO can directly interact to form peroxynitrite (ONOO^−^), nitrous oxide (NO_2_), and other RNS species [30,77,78]. In this study, drought stress disturbed the redox state homeostasis of ROS and RNS. Wang *et al.* [79] reported that ROS can trigger NO synthesis in roots of *Arabidopsis*, and it was also reported that NO also influenced ROS production and promoted the increases of ROS-scavenging enzymes in plants under stress conditions [25,80].

Drought stress-induced ROS and RNS production may be involved in pathogen infection and aflatoxin production. It has been proposed that ROS may stimulate aflatoxin production in maize and other crops [3,81], and NO may function in response to infection of *Fusarium verticillioides* in maize [82,83]. From these reports, it was implied that a complex feed-back regulation exists between ROS and RNS metabolism in plants under drought stress. In this work, ROS and RNS bursts were induced simultaneously in the tested maize genotypes, and over-accumulation of ROS and RNS caused severe oxidative and nitrosative stress on maize seedlings, which further exacerbated the visible symptoms such as wilting and necrosis. Subsequently, plants may be more susceptible to pathogen infection due to weakened defensive capabilities.

Drought stress induced a differential change of physiological and biochemical parameters in maize lines with different tolerance levels, this provides a potential link between drought stress and intensity of physiological and biochemical changes. The selection of parameters involved in these physiological and biochemical processes such as metabolites, proteins, or gene expression levels may allow them to be utilized as biomarkers for use in breeding applications [81,84].

## 4. Materials and Methods

### 4.1. Plant Materials and Growth Conditions

Six different maize inbred lines with differential sensitivity to drought stress were used for this study (Table 1). Kernels of the maize inbred lines were sown into pots (30 cm diameter, 25 cm depth, 10 kernels per pot) that were lined with a polyethylene liner, filled with locally collected field topsoil, placed in a natural lighted greenhouse, and sufficiently watered with tap water at the USDA-ARS, Crop Protection and Management Research Unit, Tifton, GA, USA. Ten days after planting (10 DAP; vegetative 2 (V2) growth stage), pots were thinned to five plants with uniform growth were left in each pot. At 30 DAP (V3/4 growth stage), pots were divided into two groups per inbred, one being normally watered (well-watered controls), while the other was subjected to progressive drought stress by withholding the water for 9 days, followed by a recovery period of normal irrigation for 3 days. During the drought treatment period, the temperature ranged from 35 to 42 °C during the day and from 22 to 28 °C at night inside the greenhouse. Soil water content was monitored to assess the stress level using the method described by Cellier *et al.* [85]. The maize seedlings were utilized for morphological, physiological and biochemical measurements from both treatments at 3, 6 and 9 DAI and 3 days of recovery (12 DAI). The experiments were conducted in two biological replicates with six and three technical replicates for each biological replicate, respectively.

### 4.2. Measurement of Plant Growth Rate and Leaf Relative Water Content

Plant growth was measured according to Chen *et al.* [41] with slight modification. At the initiation of drought treatment (0 DAI), initial plant height was measured (H_o_, height from soil surface to the base of first mature leaf), and was measured again at each treatment time point (H_e_). Plant growth (post-treatment) was then calculated as (H_e_ − H_o_). The effect of drought on plant growth was expressed as the ratio of drought treatment height increase [(H_ed_ − H_od_)/H_od_ × 100%] in comparison to the well-watered (WW) controls [(H_ew_ − H_ow_)/H_ow_ × 100%].

The changes in leaf relative water content (LRWC) under drought stressed conditions were examined at each time point also using the method reported by Chen *et al.* [41]. To measure LRWC, a leaf segment (4 cm) was sampled from the middle section of the upper, fully expanded leaves and placed into a pre-weighed and pre-labeled tube at the same time of day for each collection (9:00–10:00 am), and the fresh weight was measured after excision. The fully turgid fresh weight was measured after immersing the tissues in deionized water at 4 °C for 6 h. Dry weight was measured after drying the samples at 105 °C for 24 h. The LRWC was then calculated as (fresh weight − dry weight)/(turgid weight − dry weight) × 100%. The tube was capped immediately and then stored in a refrigerator (~4 °C). A total of six leaves per replicate were collected from six different plants.

### 4.3. Photosynthesis Parameters Measurements and Determination of Chlorophyll

The P_n_, G_s_, C_i_ and T_r_ were measured from 9:00 to 11:00 in the morning for every collection time on the leaves of four to five plants per line and treatment combination using a LI-6400XT Portable Photosynthesis System (Li-Cor, Lincoln, NE, USA) with light source (6200-02B LED, Li-Cor). The [CO_2_] in the leaf chamber was controlled by the LI-Cor CO_2_ injection system with the following chamber settings: (i) photosynthetic photon flux density of 1500 μmol·m^−2^·s^−1^; (ii) sample chamber CO_2_ held constant by the CO_2_ mixer at 400 μmol·mol^−1^ air. Leaf chlorophyll content was then measured using a portable chlorophyll meter (SPAD-502, Minolta, Tokyo, Japan).

### 4.4. ABA and IAA Content Measurement in Leaves

The ABA and IAA content assays were carried out using an ELISA-based method with the Phytodetek ABA and IAA test kit (Agdia, Elkhart, IN, USA) according to the manufacturer’s instructions and Jiang *et al*. [37]. Briefly, for the ABA assay, leaf tissues were ground in liquid nitrogen and extracted in 80% methanol with 10 mg/L butylated hydroxytoluene (BHT), and 50 mg/L citric acid in the dark for 16 h with shaking at 4 °C. The supernatant was collected after centrifugation at 4000 rpm for 20 min and diluted 10-fold with 50 mM Tris, 1 mM MgCl_2_, 150 mM NaCl, pH 7.5 for use with the Phytodetek ABA test kit. For the IAA content assay, IAA from leaf tissues was extracted and methylated by 2 M trimethylsilyl-diazomethane in hexane, and incubating for 30 min. The samples were dried and re-suspended in 500 μL 10% methanol solution. The IAA content was determined using a plate reader (Titertek Multiskan photometer, Titertek Instruments Inc., Winooski, VT, USA).

### 4.5. Superoxide Radical Staining and Quantification

The *in situ* detection of superoxide radical was done using nitroblue tetrazolium (NBT) (BP108-1, Fisher Scientific Inc., Pittsburgh, PA, USA) as a substrate according to the method of Jabs *et al.* [86] and Rao *et al.* [87] with modification as follows. Leaves were immersed in 12 mL NBT staining solution (6.0 mM NBT in 10 mM potassium phosphate containing 10 mM NaN_3_) in a 15 mL falcon tube and sand infiltrated using a vacuum pump for 20 min (KNF Neuberger, Inc., Trenton, NJ, USA). The samples were then illuminated using a light box (Model No. 300, 60 Hz, Wolf X-Ray Corp., Valdosta, GA, USA) for 8 h. After infiltration and illumination, the stained leaves were bleached in a solution of acetic acid-glycerol-ethanol (1:1:3) (*v*/*v*/*v*) at 100 °C for 2 h, and then stored in a glycerol-ethanol (1:4) (*v*/*v*) solution. Leaves were photographed under uniform lighting. Superoxide quantification was conducted according to previously described methods [88,89]. Briefly, the stained leaves were ground in liquid nitrogen and solubilized in 2 M KOH-DMSO (1/1.16) (*v*/*v*) for 4 h, and then centrifuged for 30 min at 20,000× *g*. The absorbance at 630 nm was measured using a plate reader (Titertek Multiskan photometer, Titertek Instruments Inc., Huntsville, AL, USA). Experiments were repeated three times on at least three leaves.

### 4.6. Hydrogen Peroxide Staining and Quantification

The *in situ* detection of hydrogen peroxide is conducted by staining with 3,3′-diaminobenzidine tetrahydrochloride hydrate (DAB) (AC11209-0050, ACROS Organics, Pittsburgh, PA, USA) using previously described methods with slight modification [90,91,92]. For staining, 3–5 leaves from each time point were sampled and immediately placed in 15 mL falcon tubes. DAB staining solutions were freshly prepared by adding 50 mg DAB to 47.5 mL H_2_O in a tube covered with aluminum foil, and, with constant stirring, the pH was reduced to 3.0 with 0.2 M NaOH prior to adding 25 μL Tween 20 and 2.5 mL 200 mM Na_2_HPO_4_. The staining solution was applied to the leaves in the tubes, and the volumes were adjusted to ensure that the leaves were completely immersed. The leaves were gently infiltrated under a vacuum for 20 min. The tubes were then covered with aluminum foil, and incubated for 4 h with gentle shaking (80 rpm) at room temperature in the dark. Following incubation, the staining solution was replaced with bleaching solution (ethanol:acetic acid:glycerol [3:1:1]), and the tubes were placed in 100 °C water bath for about 2 h until the chlorophyll was bleached out, and then stored in a glycerol-ethanol (1:4) (*v*/*v*) solution. The leaves were then photographed on a plain white background under uniform lighting. The H_2_O_2_ content was measured using the method reported by Kotchoni *et al.* [93]. Briefly, the DAB-stained leaves were ground in liquid nitrogen, and was homogenized in 0.2 M HClO_4_. The homogenate was then centrifuged for 10 min at 20,000× *g*. The absorbance of the supernatant at 450 nm was measured using a plate reader (Titertek Multiskan photometer, Titertek Instruments Inc., Huntsville, AL, USA).

### 4.7. Extraction and Activity Determination of SOD and CAT

Crude protein/enzyme extraction was performed in accordance with Ramel *et al.* [94] with slight modification. Leaf tissues were ground in liquid nitrogen and suspended in 5 mL of 50 mM phosphate buffer (pH 7.8) containing 0.5% (*w*/*v*) polyvinylpyrrolidone (PVP-40), 0.1% (*v*/*v*) TritonX-100, 1 mM EDTA (pH 7.4) and a mixture of protease inhibitors (1 mL per 30 g plant tissues) (P9599, Sigma-Aldrich, St. Louis, MO, USA) for 20 min at room temperature. Homogenates were centrifuged at 4000× *g* for 20 min at 4 °C. Supernatants obtained were used for enzyme activity determination. Concentrations of the protein extracts were determined using a Bradford protein assay kit (Bio-Rad, Hercules, CA, USA) with bovine serum albumin (BSA) as a standard.

The SOD activity was measured using the method reported by Beauchamp and Fridovich [95]. Briefly, a enzyme extract was mixed with 50 mM potassium phosphate buffer (pH 7.5), 130 mM methionine, 750 µM NBT, 20 µM riboflavin, and 100 µM EDTA, and the reaction mixtures were illuminated under a Transilluminator (115 V, 60 Hz, VWR TW-26, VWR Scientific, Valdosta, GA, USA) at room temperature for 20 min. The CAT activity was measured with Spectro UV-Vis Auto UV-2602 (Labomed Inc., Culver City, CA, USA) at 240 nm by measuring the digestion of H_2_O_2_ (E = 39.4 mM^−1^·cm^−1^) in 50 mM potassium phosphate buffer (pH 7.5) with protein extract as described by Aebi [96].

### 4.8. Detection and Quantification of NO

Nitric oxide (NO) was visualized using 4-amino-5-methylamino-2′,7′-difluorofluoroscein diacetate (DAF-FM diacetate; D-23844, Invitrogen, Carlsbad, CA, USA), based on the method used previously by Yang *et al.* [25]. Leaf segments were immersed and vacuum infiltrated with 10 µM DAF-FM DA in 50 mM Tris-HCl buffer (pH 7.4). Infiltrated leaf segments were then incubated for 2 h at 37 °C, then fluorescence was detected using a Zeiss SV epi-fluorescence stereomicroscope with a 480 ± 30 nm excitation filter and a 515 nm emission filter (Chroma Technology, Brattleboro, VT, USA) coupled with a Zeiss Axiocam digital camera. The relative quantification of NO in leaves was conducted using DAF-FM DA according to published protocols [97,98]. Leaf segments of approximately 12 mm^2^ were incubated with 10 μm DAF-FM diacetate in 10 mM Tris-HCl (pH 7.5) in 96-well plates, and were then washed in the same buffer for 30 min. Fluorescence measurements were obtained on a fluorescent plate reader (Synergy HTX Multi-Mode Reader, Biotek, Winooski, VT, USA). Fluorescence intensity was calculated by subtracting the DAF fluorescence measured in leaves from wells with Tris-HCl buffer only. The staining procedure for each sample was conducted with three replicates.

### 4.9. Determination of the NOS and GSNOR Activities

The NOS activity was measured using a Nitric Oxide Synthase Assay Kit (EMD Millipore, Billerica, MA, USA) based on the manufacturer’s instructions. Total protein was extracted with 100 mM HEPES-KOH (pH 7.4), 1 mM EDTA (pH 7.4), 10% glycerol (*v*/*v*), 5 mM DTT, 10 µM PMSF, 0.1% Triton X-100 (*v*/*v*), 1% polyvinylpyrrolidone (PVP-40) and 20 µM FAD. GSNOR activity was measured as described by Corpas *et al*. [27].

### 4.10. Statistical Analysis

Analysis of Variance (ANOVA), pearson correlation and clustering were performed using in-house developed R-software (R Development Core Team, Vienna, Austria). Principal component analysis was performed by “bpca” algorithm from the pcaMethods package [99]. The ANOVA analysis was conducted using Genotype, Treatment, and Tolerance as factors. The *p*-values were corrected using the method reported by Bonferroni and Yekutieli [100].

To estimate the statistical significance between means, ANOVA was performed for the entire dataset using SPSS v.15.0 (SPSS Inc., Chicago, IL, USA). The means (*n* = 6) were separated using Fisher’s Least Significant Difference (LSD) test with α = 0.05. Means not significantly different (*p* > 0.05) share the same letter in graphical representations, provided by the GraphPAD software (v.5.01, GraphPad Software Inc., San Diego, CA, USA). The data were Box-Cox transformed prior to use in PCA using the NIA array analysis tool [101]. We used the default settings for the analysis except for the false discovery rate (FDR) thresholddefined as 0.01. For the PCA analysis, the settings were as follows: covariance matrix type, principal components, one-fold change threshold for clusters, and 0.8 correlation threshold for clusters as used by Chen *et al.* [102].

## 5. Conclusions

The results in this study showed that drought stress resulted in obvious morphological and physiological changes, and induced a rapid accumulation of ROS and NO. This resulted in oxidative and nitrosative stress and activated the antioxidant defense system, primarily characterized by increases of SOD and CAT activities in all tested lines. The metabolic alterations of ROS and RNS levels were more pronounced in the sensitive lines as compared to the tolerant ones with the same being true for the activities of ROS and RNS scavenging enzymes. This implies that a much more vigorous metabolic response was likely triggered in the sensitive lines than in the tolerant lines. Taken together, these observations suggest that maize lines with contrasting drought sensitivity possess different defensive and responsive processes counteracting oxidative and nitrosative damage. However, possible synergistic effects and homeostasis of ROS and RNS in this system under drought stress conditions have yet to be elucidated and warrant further study.

## Supplementary Materials

Supplementary materials can be found at http://www.mdpi.com/1422-0067/16/10/24791/s1.
