# Supplementary material for: Stress Sensitivity Is Associated with Differential Accumulation of Reactive Oxygen and Nitrogen Species in Maize Genotypes with Contrasting Levels of Drought Tolerance
Source: Int J Mol Sci. 2015 Oct 19;16(10):24791–819. doi: 10.3390/ijms161024791 (PMC4632777; doi:10.3390/ijms161024791)
Supplement: Supplementary file 1 [file ijms-16-24791-s001.pdf]

Supplemental Information

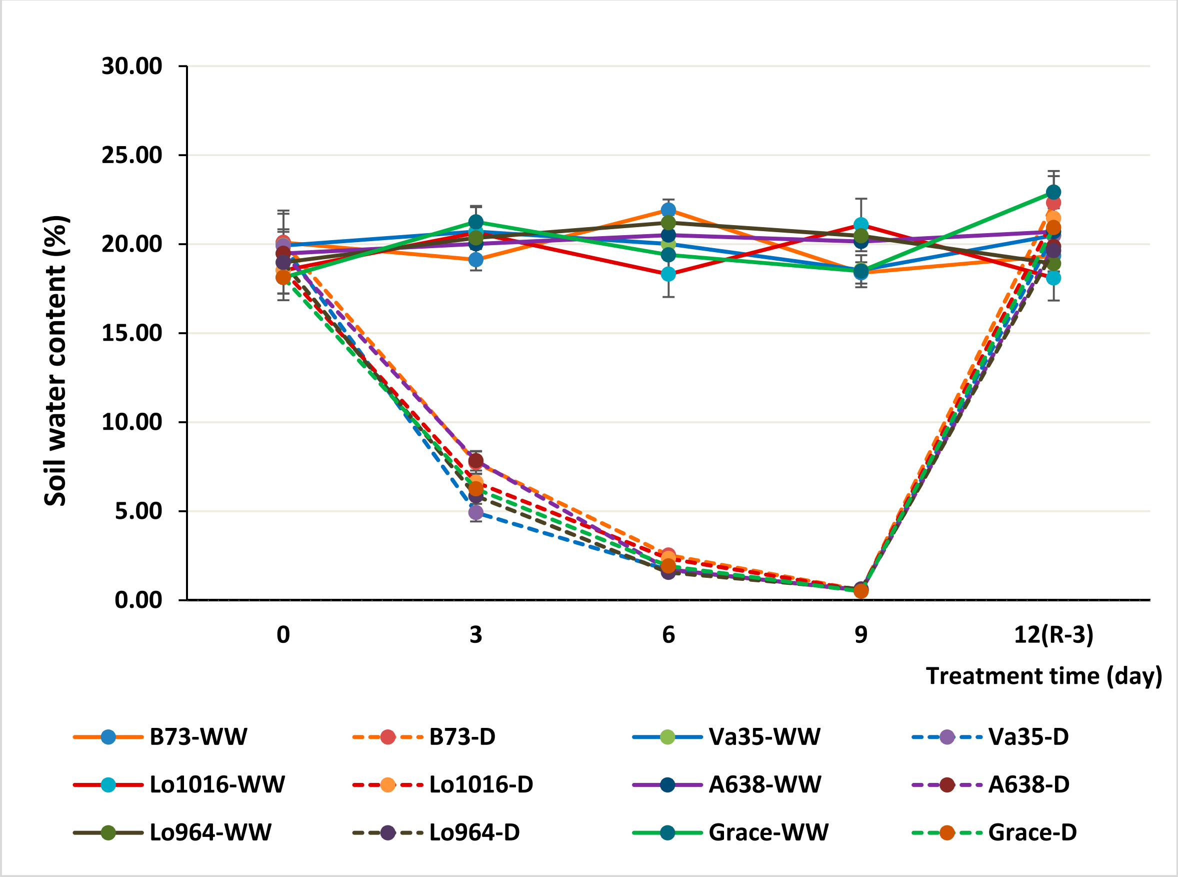

Figure S1. Soil water content at over time under well-watered (WW) and drought (D) conditions.

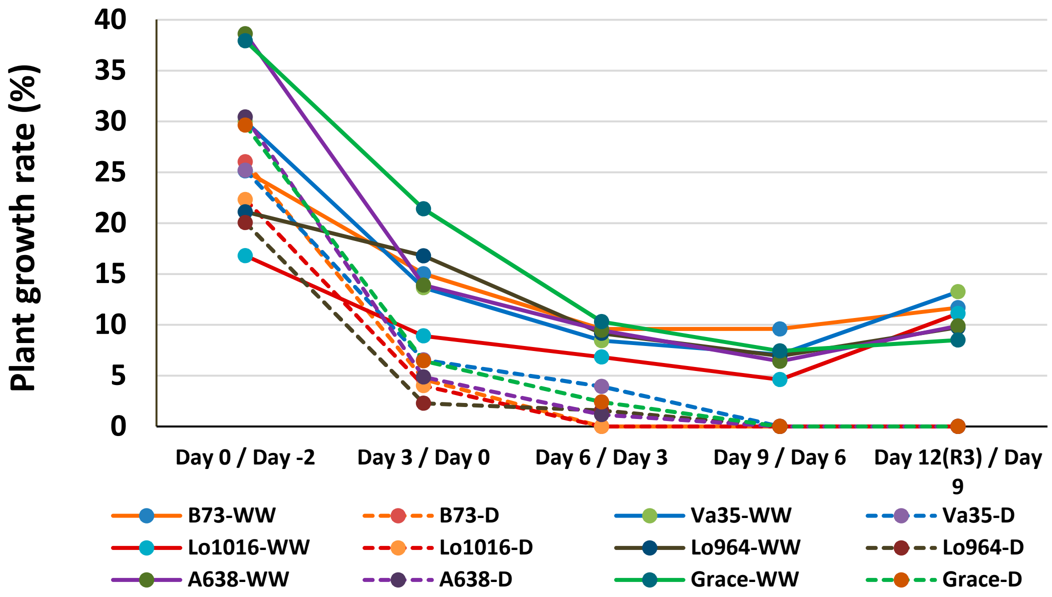

Figure S2. Growth rates of maize seedlings under well-watered (WW) and drought (D) conditions over time.

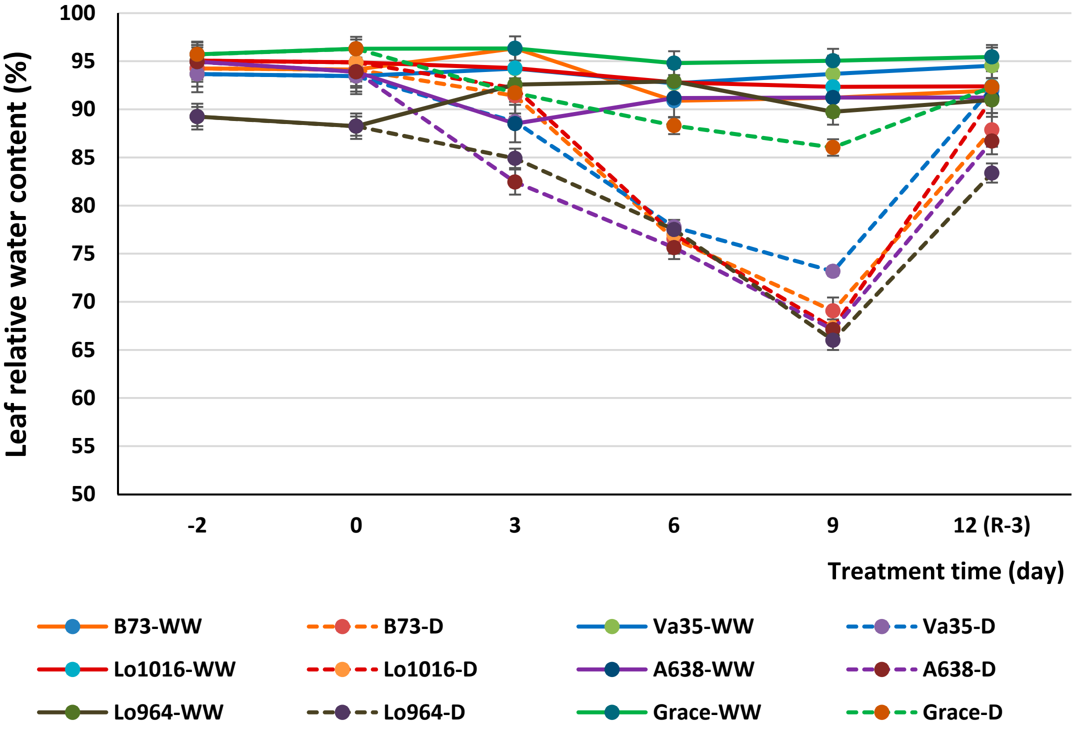

Figure S3. Leaf relative water content (LRWC) of maize seedlings under well-watered (WW) and drought (D) conditions over time.

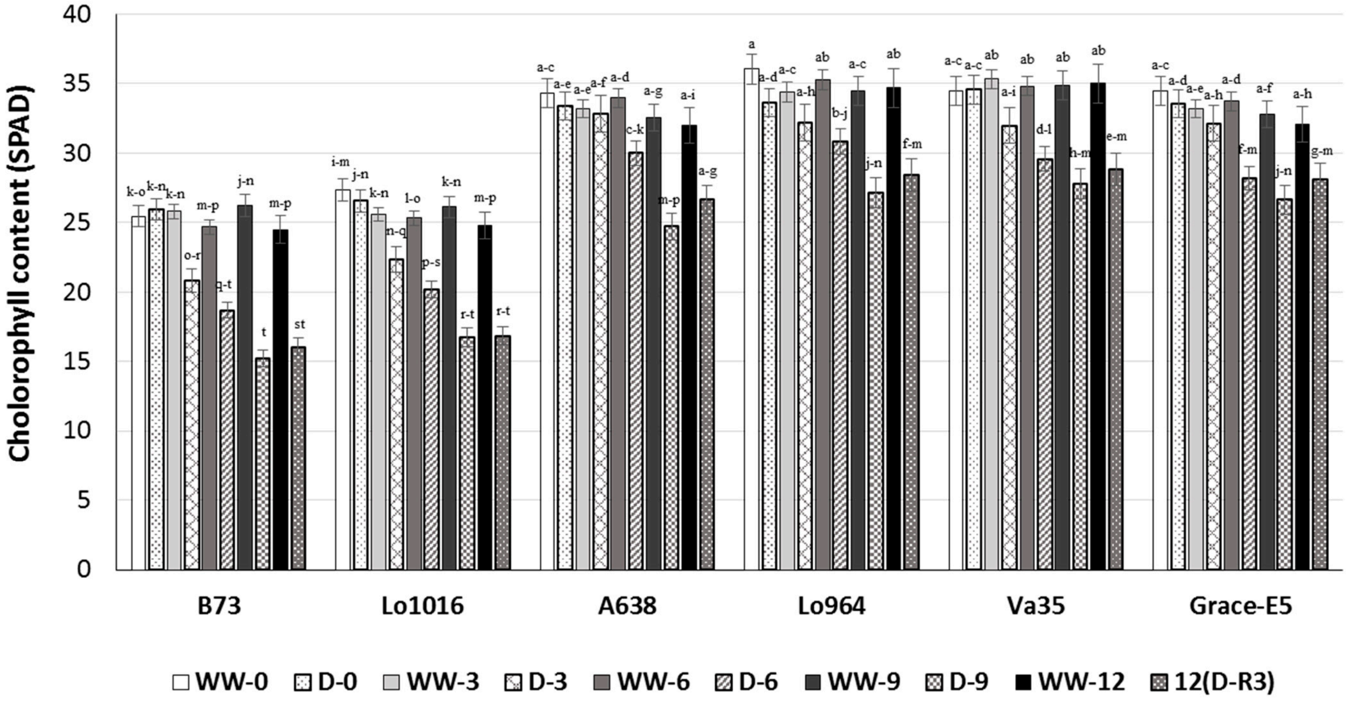

Figure S4. Chlorophyll content of maize seedling leaves. Different letters indicate significant differences ($p < 0.05$) based on Tukey's test between control and treatments and between different treatment times. Data represent the mean ± SD of three or more replicates.

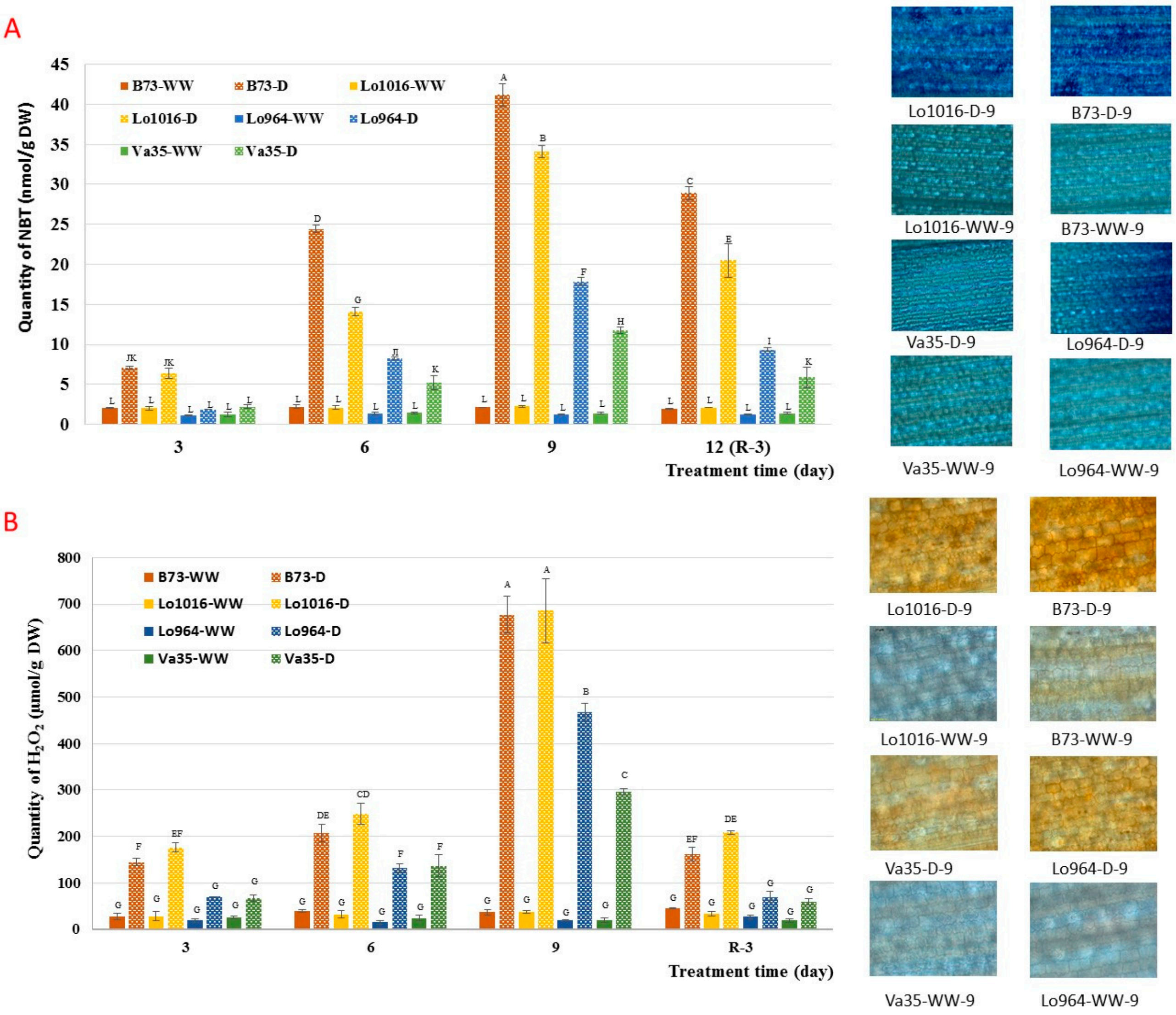

Figure S5. Quantification of superoxide radical (**A**) and hydrogen peroxide (**B**) in leaves of maize plants subjected to drought stress. WW refers to well-watered leaves; DT refers to drought treated leaves. The right figure in (**A**) or (**B**) shows the staining difference in superoxide radical or hydrogen peroxide accumulation observed under microscopy. Different letters indicate significant differences ($p < 0.05$) based on Tukey's test between control and treatments and between different treatment times. Data represent the mean ± SD of three or more replicates.

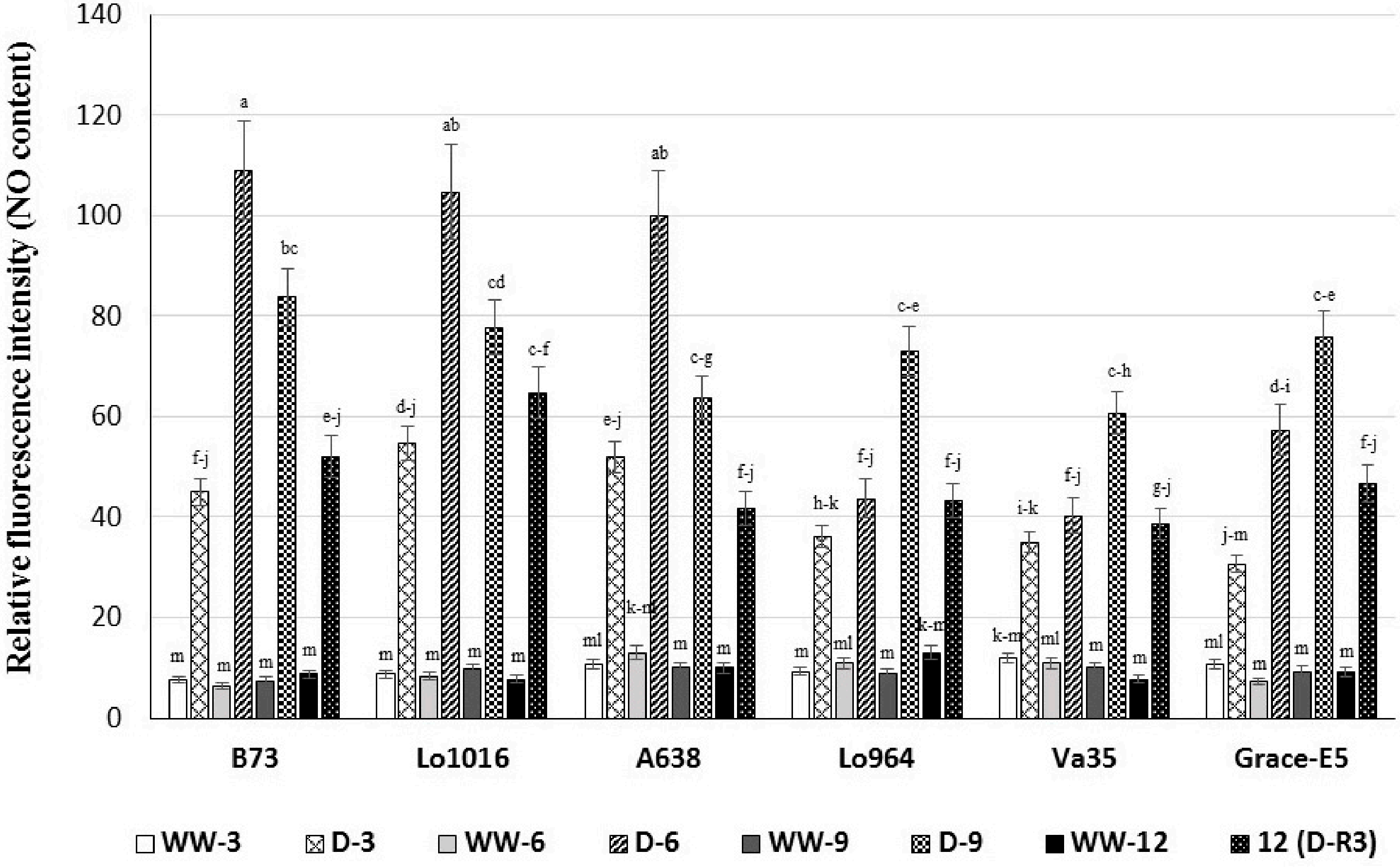

Figure S6. Quantification of nitric oxide (NO) in leaves of maize plants subjected to drought stress. WW refers to well-watered leaves, DT refers to drought treated leaves. Different letters indicate significant differences ($p < 0.05$) based on Tukey's test between control and treatments and between different treatment times. Data represent the mean ± SD of three or more replicates.

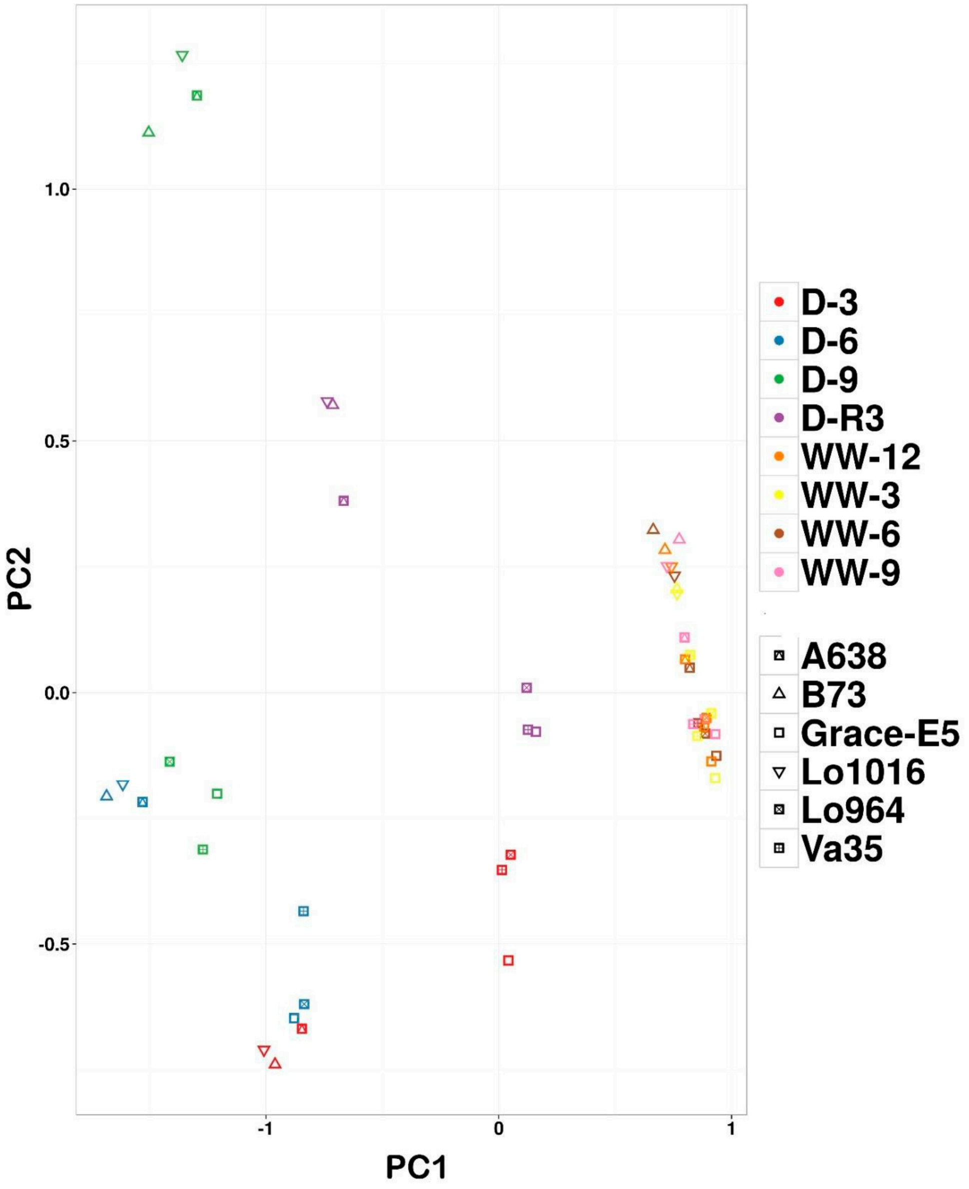

Figure S7. Time-series principal component analysis (PCA) of all test traits in six different lines under well-watered and drought conditions. WW-3, -6, -9 and -12 refers to maize plants under well- watered conditions for 3, 6, 9 and 12 days; D-3, -6, -9 and -R3 refers to maize plants subjected under drought treatments for 3, 6 and 9 days, and then recovery watering for additional 3 days.

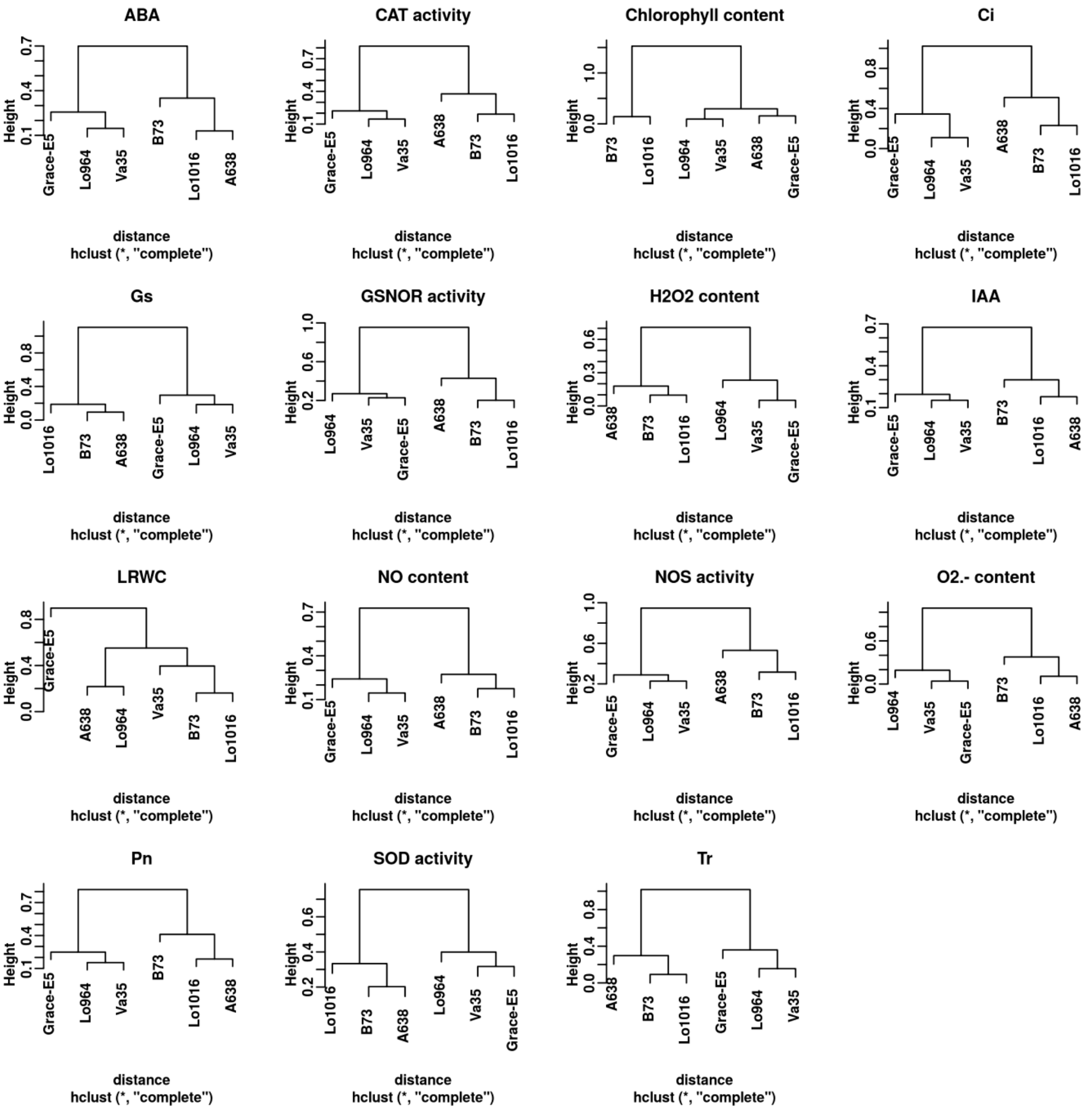

Figure S8. Time-series clustering of all tested traits in all six maize genotypes.
